# Editorial: Body composition assessment techniques in clinical and epidemiological settings: Development, validation and use in dietary programs, physical training and sports

**DOI:** 10.3389/fnut.2023.1146553

**Published:** 2023-01-31

**Authors:** Roberto Fernandes da Costa, Rossana Candiota Nogueira, Mauro Fisberg, Gerson Ferrari

**Affiliations:** ^1^Physical Education Department, Health Sciences Center, Federal University of Rio Grande do Norte, Natal, Brazil; ^2^Faculty of Health Sciences and Medicine, Bond University, Gold Coast, QLD, Australia; ^3^Pensi Institute-José Luis E. Setubal Foundation and Pediatrics Department, Federal University of São Paulo, São Paulo, Brazil; ^4^Escuela de Ciencias de la Actividad Física, el Deporte y la Salud, Universidad de Santiago de Chile (USACH), Santiago, Chile; ^5^Grupo de Estudio en Educación, Laboratorio de Rendimiento Humano, Actividad Física y Salud (GEEAFyS), Universidad Católica del Maule, Talca, Chile

**Keywords:** body composition, fat free mass (FFM), fat mass, anthropometry, dual-energy X-ray absorptiometry (DXA), bioelectrical impedance analysis (BIA), comput(eriz)ed tomography, air displacement plethsymography

Body composition assessment is essential in both clinical and field settings to accurately describe and monitor nutritional status for a variety of medical conditions and physiological processes. Patients with cancer, osteoporosis, cardiovascular disease, diabetes, as well as sick and malnourished patients, pregnant women, nursing mothers, and the elderly, are a few examples among several other diseases that can be assessed by body composition. Body composition outcomes help evaluate the effectiveness of nutritional interventions, the alterations associated with growth and disease conditions, and it contributes to the development of personalized physical training programs (1–3).

There are several techniques for assessing body composition, from simple body indices based on anthropometric measurements to sophisticated laboratory methods such as magnetic resonance imaging (4), with the ability to assess different body compartments at different levels (5, 6). Thus, many studies have been conducted in order to develop and validate techniques, which can be extremely useful for health professionals to estimate body composition components such as fat mass, muscle mass, bone mass, and residual mass, or simply fat mass and fat-free mass (7–10).

The aim of this Research Topic is to address the most recent innovations in body composition assessment for its application in epidemiological studies, as well as in clinical practice, providing health professionals with concepts and evidence of its usefulness, while assisting them with the most appropriate selection of techniques according to the characteristics of the individuals or groups evaluated.

In this Research Topic, 22 papers were published, divided into three groups of studies: development of predictive models and validation of existing predictive models; cross-sectional descriptive studies; intervention studies; and a systematic review and meta-analysis.

All studies used anthropometry and/or bioelectrical impedance as a technique to assess body components, which can be explained by the relatively low cost and high applicability in clinical and field conditions (2, 11). However, studies that developed or tested the validity of predictive models of these techniques used mainly dual-energy X-ray absorptiometry (DXA) as the standard technique, while two studies used computed tomography and one study used air displacement plethysmography.

The most discussed aspect was the development of predictive models and the validation of existing models. Nine of the 22 (40.9%) published articles covered this topic, which represents a vital role for studies in body composition assessment, as the mathematical models developed to estimate body components are more specific with the source population, indicating that they cannot be generalized to several populations (12).

The study by Costa et al. first tested the validity of eight equations for estimating fat-free mass (FFM) by bioelectrical impedance analysis, developed for adolescents from different populations, verifying that none of them met the validity criteria in the sample of adolescents aged 10 to 19 years, from the northeastern region of Brazil. Thus, the authors developed and cross-validated a specific mathematical model for this population. Still, in the same region of the country, but for adults aged 20 to 59 years, Ribeiro da Costa et al. tested the validity of the body adiposity index (BAI) proposed by Bergman et al. (13), finding low validity for the studied sample. Then, the authors developed a regression equation that was included in the model, in addition to the BAI variables (height, waist circumference, and hip circumference), weight, gender, and age, to estimate the FFM and total body fat, using anthropometric measurements.

Likewise, or more important than the amount of body fat, is its distribution, as a higher concentration of fat in the abdominal region, especially visceral fat, is associated with non-communicable chronic diseases and increased morbidity and mortality (14, 15). However, measuring fat in this region demands high-cost laboratory techniques, such as magnetic resonance imaging or computed tomography (11, 16), indicating the need for valid predictive models for clinical or epidemiological screening. This aspect was contemplated in two articles by Lai et al., who developed an equation for the abdominal subcutaneous fat area using bioimpedance analysis (BIA) combined with a sagittal abdominal diameter, and of Ji et al., who developed formulas for calculating L3 skeletal muscle mass index and visceral fat area based on simple anthropometric measurements. Both studies used computed tomography as the standard technique.

Another aspect worth mentioning is that the validity of techniques for estimating the body composition of under 6 year-old children still needs to be clarified in the literature (17). Lyons-Reid et al., using air displacement plethysmography as a reference, developed empirical prediction equations to estimate FFM in childhood. The authors demonstrated that the inclusion of impedance in the equations instead of just anthropometric parameters improved performance in most cases, but the difference was slight. Further investigation was suggested before the routine use of BIA in childhood can be recommended.

Studies on changes in body composition due to aging have been highlighted, mainly due to the negative impact of sarcopenia on health in elderly populations, suggesting the need for valid clinical techniques to assess this condition (18). Of the four studies published in this issue which proposed testing techniques' validity, three included a sample composed of older adults, using bioimpedance and/or anthropometry. Cáñez-Ríos et al. verified the agreement between six bioimpedance equations and DXA to estimate the appendicular skeletal muscle mass; van den Helder et al. validated bioimpedance analysis to diagnose low appendicular lean mass; and Velázquez-Alva et al. evaluated the agreement between bioimpedance measurements and five anthropometric equations for estimating body fat, using DXA as a standard.

Another important aspect is the difficulty of assessing body composition in people with disabilities. Although there are mathematical models for estimating fat-free mass, by bioelectrical impedance analysis, in people with spinal cord injury, it needs to be clarified whether they can be generalized to people with this condition (19). Bauermann et al. demonstrated that using non-specific impedance measurement equations can lead to an erroneous interpretation of FFM values in male subjects with spinal cord injury, indicating the need to develop new predictive equations for this group.

Regarding the cross-sectional descriptive studies (*n* = 7), two articles addressed the Phase Angle, a variable obtained through bioelectrical impedance testing that has been widely used as a marker of cell membrane integrity and a prognostic factor in several diseases (20, 21). Mattiello et al., produced Phase Angle percentile curves in a healthy population covering most of the life cycle, stratified by sex and age, using generalized additive models for location, scale, and shape as a continuous function of age. de Moraes et al., studying adolescents, demonstrated that the variability in the phase angle is related to interindividual variation in sex, age, and maturation status, as well as differences in body size. The authors concluded that research with adolescents considering phase angle should use multilevel modeling with standardized parameters as default to adjust for the concurrent influence of sex, age, maturity status, and body size.

Using anthropometric measurements, such as body mass, height, body circumferences, and indices based on these and other measures derived from bioelectrical impedance analysis constitutes a tool for risk screening for adverse health conditions throughout life (13, 22). These measurements or indices may be associated with arterial properties and variations (Gómez-García et al.); the lipid and glucose profile of children and adolescents (Nogueira-de-Almeida et al.); malnutrition and its repercussions for all-cause mortality and cardiovascular mortality (Fan et al.); high blood pressure in adolescents (Borges et al.); and hypertensive disorders of pregnancy (Yuan et al.).

Many intervention studies with dietary and/or physical exercise programs seek to demonstrate their impact on changes in body composition (23, 24). In this Research Topic, five articles performed interventions to analyze different outcomes. Sheikholeslami-Vatani and Rostamzadeh investigated the effect of 8 weeks of high-intensity interval training and vitamin D3 supplementation on changes in appetite-dependent hormones and body composition in sedentary overweight men, finding satisfactory results. In the study by Lazzer et al., a 3-week multidisciplinary body weight reduction program with moderate energy restriction and regular physical activity was sufficient to determine a 4–5% reduction in body mass, in addition to improving physical activity and induce beneficial changes in body composition in obese adolescents and adults. They carried out a randomized controlled trial to test the effects of aquatic resistance training and dietary education on health indicators in older women, including body composition. The results suggest that older women who practice regular and programmed underwater resistance training, among other benefits, have improved body composition variables (smaller fat compartments and greater muscle mass).

Another randomized controlled trial aimed to verify the impacts of water supplementation on body composition indices in young adults after a 12-h overnight fast to determine the ideal volume of water to improve body water composition. Among other findings, the authors concluded that 200 mL was the minimum volume capable of improving the distribution of water content among the participants of this study (Zhang et al.). And finally, studying preterm-born preschoolers with very low birth weight, Fernandes et al. verified the impact of a continuous early home-based intervention program on body composition. The study showed that an early intervention protocol from the newborn intensive care unit (NICU) to a home program performed by mothers of preterm with very low birth weight (VLBW) children from low-income families has a small effect on fat-free mass.

As mentioned, this Research Topic also published a systematic review and meta-analysis that surveyed diagnostic studies to identify the optimal cutoff value for the waist-to-height ratio (WHtR) to predict central obesity in children and adolescents. The 12 articles included in the meta-analysis led to the conclusion that 0.49 was the best cutoff point to predict abdominal obesity in children and adolescents of both sexes.

In summary, the results of the studies and the review in this volume bring a substantial amount of relevant data on body composition assessment techniques in their different uses. Thus, these manuscripts contribute to a better understanding and better using different techniques for estimating body components in clinical and field situations to optimize dietary and physical exercise programs.

## Author contributions

All authors participated in the elaboration, writing, revision and approval of the final document of this editorial.
